# Fluid lavage in patients with open fracture wounds (FLOW): an international survey of 984 surgeons

**DOI:** 10.1186/1471-2474-9-7

**Published:** 2008-01-23

**Authors:** Brad Petrisor, Kyle Jeray, Emil Schemitsch, Beate Hanson, Sheila Sprague, David Sanders, Mohit Bhandari, the FLOW Investigators

**Affiliations:** 1 Department of Surgery, McMaster University, Hamilton, Ontario, Canada; 2 Department of Surgery, Greenville Hospital System, Greenville, South Carolina, USA; 3 Department of Surgery, University of Toronto, Toronto, Ontario, Canada; 4 AO Foundation, Davos, Switzerland; 5 Departments of Epidemiology and Biostatistics, McMaster University, Hamilton, Ontario, Canada; 6 Department of Surgery, University of Western Ontario, London, Ontario, Canada

## Abstract

**Background:**

Although surgeons acknowledge the importance of irrigating open fracture wounds, the choice of irrigating fluid and delivery pressure remains controversial. Our objective was to clarify current opinion with regard to the irrigation of open fracture wounds.

**Methods:**

We used a cross-sectional survey and a sample-to-redundancy strategy to examine surgeons' preferences in the initial management of open fracture wounds. We mailed this survey to members of the Canadian Orthopaedic Association and delivered it to attendees of an international fracture course (AO, Davos, Switzerland).

**Results:**

Of the 1,764 surgeons who received the questionnaire, 984 (55.8%) responded. In the management of open wounds, the majority of surgeons surveyed, 676 (70.5%), favoured normal saline alone. Bacitracin solution was used routinely by only 161 surgeons (16.8%). The majority of surgeons, 695 (71%) used low pressures when delivering the irrigating solution to the wound. There was, however considerable variation in what pressures constituted high versus low pressure lavage. The overwhelming majority of surgeons, 889 (94.2%), reported they would change their practice if a large randomized controlled trial showed a clear benefit of an irrigating solution – especially if it was different from the solution they used.

**Conclusion:**

The majority of surgeons favour both normal saline and low pressure lavage for the initial management of open fracture wounds. However, opinions varied as regards the comparative efficacy of different solutions, the use of additives and high versus low pressure. Surgeons have expressed considerable support for a trial evaluating both irrigating solutions and pressures.

## Background

Approximately 3–4% of all fractures are open fractures [1]. This translates to over 3,400 open fractures in Canada and an estimated 250,000 open fractures in the United States annually[1,2]. Reported rates of infection following severe open tibial fractures have ranged from 5% to 50% with infection potentially resulting in a significant number of repeat operations, delay in fracture healing and possible development of fracture nonunion [3-7]. Prevention of infection, stable fixation of the fractured bone and restoration of patient function remain paramount in the management of these complex injuries.

Meticulous debridement of contaminated soft tissues along with copious fluid irrigation is regarded as the most important initial step in the surgical wound management of open fractures [2,4,8-10]. While several authors have proposed techniques for irrigation, many issues remain unresolved including the irrigating pressures, the amount of irrigation, and the irrigating solution[2,11-14]. Identifying the optimal irrigating solution and pressure at which this solution should be delivered has global relevance. By 2020, disability from traffic accidents, the major cause of fractures is estimated to rank in the top 3 of all cause disability from disease. Accelerated urbanization and industrialization in India and China, which represent 40% of the world's population, have resulted in an alarming increase in traumatic injuries. A vehicular accident is reported every three minutes and a death every ten minutes on Indian roads. For every death, 3 patients survive and live with disability.

To explore current practice in the management of open wounds, we conducted an international survey of practicing orthopaedic surgeons in order to learn about their preferences for fluid irrigation of open fractures and to identify the need for future research in this area. We hypothesized that there was considerable variability in the operative treatment of open fracture wounds. Furthermore, we reasoned that the results of this survey may identify factors that influence a surgeon's preference for a particular treatment, serve to educate the orthopaedic community on issues regarding the treatment of open fractures, and allow for the development of future clinically related trials, which could help resolve the current controversy on optimal irrigation techniques among orthopaedic surgeons.

## Methods

### Questionnaire Development

#### Item Generation

We developed a questionnaire using focus groups, key informants, and the previous literature. Orthopaedic surgeons in Canada and the United States participated in the development of the questionnaire.

The items generated from the focus group were improved by data from a MEDLINE search of articles published from 1966 to 2006 using text words "irrigation," "debridement," "infection," "open fractures," "pressure," "anti-bacterial agents," "detergents," "soaps," "solutions," "disinfection," and "bacitracin." Further items were generated with key informants. Surgeons specializing in orthopaedic trauma provided additional input into potential items for the questionnaire. We used a "sample to redundancy" approach by which we contracted new surgeons until no new items for the questionnaire emerged.

#### Pretesting and Validity Assessments

We pretested the questionnaire with an independent group of four orthopaedic surgeons to evaluate whether the questionnaire as a whole appeared to adequately address the question of current practice in treating open fractures (face validity); and whether the individual questions adequately reflected the four broad domains of surgeon training and experience, technical aspects of irrigation and debridement, and perioperative issues in open fractures (content validity). These surgeons also commented on the clarity and comprehensiveness of the questionnaire.

The final questionnaire framed the response options in one of two ways: five-point Likert scales or nominal scales. A previous report has shown that closed-ended questions resulted in fewer incomplete questionnaires than open-ended formats[15]. We obtained information regarding surgeon age, sex, number of open fractures treated per year, supervision of resident trainees, continent of practice, fellowship training in trauma, and type of practice (community or academic). Academic practice was defined as a formal affiliation with a university centre. In addition, we asked surgeons tochoose their preference for the following: time point for wound management, location of initial irrigation and debridement, time point to repeat debridement, type of irrigating solution, amount of irrigating solution, pressure (high vs. low) used, method of delivering irrigating solution, and definitions of high pressure and low pressure.

### Questionnaire Administration

We surveyed all surgeons who were members of the Canadian Orthopaedic Association (COA) (active members, associate members, international members, senior members, honourary members, and emeritus members). Members of the COA each received a mailed package that included a copy of the survey, a personalized cover letter, and a return envelope. Surveys were not sent to non-responders, as repeat mailings were not necessary to obtain our required sample size. In addition, we surveyed orthopaedic surgeons attending an international trauma course (Davos, Switzerland). These surgeons each received a cover letter and copy of the survey to complete while attending the trauma course.

No monetary incentives or pre-notification telephone calls were used for this survey. Individual responses were kept confidential and questionnaire completion was voluntary. Individual responses were kept confidential and questionnaire completion was voluntary. This study was approved by our local ethics review board.

### Sample Size

In order to determine the number of respondents needed to sufficiently power our analysis, we assumed, based on findings reported in the literature, that approximately 35% of surgeons surveyed used high pressure irrigation. In addition, we intended to use a 95% confidence interval, with a 95% confidence level. The calculation for appropriate sample size was performed according to the following formula:

N = (Z_α_/w)^2^p(1 - p)

Where:

Z = z value (1.96 for 95% confidence interval)

w = the confidence interval, expressed as decimal (0.05 = +/- 5)

p = percentage picking a choice, expressed in decimal (35% = 0.35)

According to our calculation, approximately 650 completed questionnaires facilitated meaningful analysis. Based on previous surveys of orthopaedic surgeons we anticipated a 70% response rate; therefore, a total of at least 930 questionnaires needed to be administered to obtain the desired number of completed surveys.

### Statistical Analysis

We summarized categorical and dichotomous variables with percentages. Whenever the distribution of responses for a particular item in the questionnaire had multiple empty cells (cutpoints), we collapsed the categories in that particular item to achieve an even distribution of responses.

We performed univariable linear regression analysis to evaluate association between surgeon age, type of practice, fellowship training, trauma volume, amount of irrigation and type of irrigation used. Factors that were significantly associated with each dependent variable were combined in a multiple regression analysis. We plotted residuals from the regression analysis to ensure a normal distribution of the data points. We reported beta values and their 95% confidence intervals for each independent variable in the analysis.

## Results

### Characteristics of the respondents

Of the 1,764 surgeons who received the questionnaire, 984 (55.83%) responded. Of the 764 COA members, 328 (42.9%) completed the survey. Of 1,000 participants who received the survey at the trauma course, 656 responded by completion (65.6%). The typical respondent was a male over 30 with greater than 5 years of practice (Table 1). The majority [597 (61.2%)] worked in an academic centre and supervised trainees [587 (59.9%)] (Table 1). Two hundred and ninety-eight (37%) respondents had completed further orthopaedic trauma fellowship training with 344 (35.3%) of respondents spending >50% of their practice doing orthopaedic trauma (Table 1). Six hundred and three (63%) respondents see more than 10 open fractures per year and 107 (11.2%) see over 50 open fractures per year.

**Table 1:** Demographics

**Characteristic**		**No. (%)**
Age	Less than 30	95 (9.7%)
	30–40	403 (41.0%)
	41–50	278 (28.3%)
	51–60	147 (15.0%)
	Over 60	59 (6.0%)
Number of years in practice	Less than 5	247 (25.6%)
	5–10	261 (27.1%)
	11–15	158 (16.4%)
	16–20	108 (11.2%)
	Over 20	189 (19.6%)
Type of hospital	Academic (University Affiliated)	597 (61.2%)
	Non-Academic	378 (38.8%)
Supervise residents in training	Yes	587 (59.9%)
	No	393 (40.1%)
Completed a fellowship in trauma	Yes	298 (30.6%)
	No	677 (69.4%)
Proportion of practice with orthopaedic trauma	0–25%	306 (31.4%)
	26–50%	324 (33.3%)
	51–75%	220 (22.6%)
	76–100%	124 (12.7%)
Number of open fractures treated per year	0	14 (1.5%)
	1–10	339 (35.5%)
	11–20	245 (25.6%)
	21–30	149 (15.6%)
	31–40	68 (7.1%)
	41–50	34 (3.6%)
	>50	107 (11.2%)

#### Management Preferences

##### Type of Fluid

When asked what type of irrigating solution was routinely used for open fracture wounds in their practice (Table 2), 676 (70.5%) respondents favoured normal saline alone. The most common additive to normal saline was bacitracin antibiotic solution chosen by 161 (16.8%) respondents. Fifty-five (6%) respondents used an iodine based solution and only 12 (1.3%) respondents used a soap, or detergent, when irrigating open wounds. Over half the surgeons believed that neither bacitracin nor soap solution provided additional efficacy over saline alone [453 (50.1%) and 489 (55.1%), respectively] (Table 3). Alternatively, the majority of surgeons believed iodine and chlorhexidine were more effective than saline alone [398 (44%) and 413 (46.3%), respectively] (Table 3). Surgeons who used only normal saline solution were significantly more likely to operate on more open fractures yearly (odds ratio = 1.1, p = 0.048), worked in an academic hospital setting (Odds ratio = 1.3, p = 0.04) and believed that bacitracin was no more effective than saline alone (Odds ratio = 2.9, p < 0.001)

**Table 2:** Type of Irrigation Solution Routinely Used

**Irrigation Solutions**		**No. (%)**
Irrigation solution routinely used for wound management	Normal saline alone	676 (70.5%)
	Normal saline with bacitracin (or equivalent antibiotic solution)	161 (16.8%)
	Soap solution (or equivalent detergent)	12 (1.3%)
	Chlorhexidine	15 (1.6%)
	Iodine based	55 (5.7%)
	Hydrogen peroxide	12 (1.3%)
	Other	28 (2.9%)

**Table 3:** Effectiveness of Solutions Relative to Normal Saline in Reducing Infection Risk

**Solution**	Definitely **less **effective than Saline	Moderately **less **effective than Saline	Equivalent to Saline	Moderately **more **effective than Saline	Definitely **more **effective than Saline
**Bacitracin**	33 (3.7%)	62 (6.9%)	453 (50.1%)	300 (33.2%)	56 (6.2%)
**Soap solution**	84 (9.5%)	131 (14.8%)	489 (55.1%)	161 (18.1%)	23 (2.6%)
**Iodine solution**	60 (6.6%)	105 (11.6%)	342 (37.8%)	307 (33.9%)	91 (10.1%)
**Chlorhexidine solution**	51 (5.7%)	85 (9.5%)	342 (38.4%)	313 (35.1%)	100 (11.2%)

##### Amount of irrigating solution

For Type 1 open injuries the majority of surgeons, 609 (63.9%), preferred 3 litres or less of irrigating solution. For Type II open wounds, 475 surgeons preferred between 3 and 6 litres of irrigating fluid (50.1%). In Type III open wounds, the most commonly endorsed volume by 386 (41.3%) surgeons remained between 3 and 6 litres of irrigating fluid, surprisingly not 9 or more liters. However, the respondents were 8-fold more likely to use more than 9 litres of irrigating solution as the Gustilo Type increased from Type I to Type III (p < 0.01) (Table 4).

**Table 4:** Amount of Irrigation Solution Routinely Used

**Fracture Type**	**<3 L**	**3–6 L**	**7–9 L**	**>9 L**
**Type I**	609 (63.9%)	277 (29.1%)	44 (4.6%)	23 (2.4%)
**Type II**	255 (26.9%)	475 (50.1%)	157 (16.6%)	61 (6.4%)
**Type III**	114 (12.2%)	386 (41.3%)	250 (26.7%)	185 (19.8%)

##### Irrigating Pressure

Six hundred and ninety-five surgeons (71%) preferred the delivery of irrigating fluid at low pressure. The majority of whom [317 surgeons (32.2%)] did so with a bulb syringe (Table 5). However, there was some discrepancy in defining both high and low pressure lavage (Table 6). Surgeons definitions about high pressure lavage varied considerably, while the majority [609 (73.1%) surgeons] agreed that low pressure represented pressures less than 20 p.s.i. (Table 6). Regression analysis suggested that younger surgeon age (Odds ratio = 1.7, p = 0.049) and the belief that high pressure irrigation on fractures may delay fracture healing (Odds ratio = 2.1, p < 0.001) were significantly associated with surgeon preferences favoring lower pressure irrigation.

**Table 5:** Types of Pressure – High versus Low

			**No. (%)**
Preferred method of delivering irrigating solution to wounds	*High Pressure*	Battery operated/pulsatile	345 (35.1%)
		Other	16 (1.6%)
	*Low Pressure*	Battery operated irrigation device	176 (17.9%)
		Manual irrigation via bulb syringe	317 (32.2%)
		Gravity Flow irrigation via tubing	175 (17.8%)
		Gravity Flow irrigation dispensed via a basin/bowl	20 (2.0%)
		Other	7 (0.7%)

**Table 6:** Definitions of High and Low Pressure

**Type**	**Amount**	**No. (%)**
Define high pressure in pounds/square inch (psi)	0–20 psi	26 (3.2%)
	21–40 psi	297 (36.5%)
	41–60 psi	312 (38.4 %)
	61–70 psi	135 (16.6%)
	>70 psi	43 (5.3%)
Define low pressure in pounds/square inch (psi)	0–20 psi	609 (73.1%)
	21–40 psi	200 (24.0%)
	41–60 psi	13 (1.6%)
	61–70 psi	11 (1.3%)
	>70 psi	0

##### Need for Further Research

There was considerable support among respondents for a clinical trial evaluating outcomes following both the use of different irrigating solutions as well as irrigating pressures [803 (84.8%) and 730 (77.6%) respectively] (Tables 7 and 8). The overwhelming majority of surgeons [889 (94.2%)] reported they would change their practice if a large randomized controlled trial showed a clear benefit of an irrigating solution – especially if it was different from the solution they used. Table 8 demonstrates the relative risk reduction of infection, in percent, needed to change the surgeons current practice pattern. And 19.3%, the largest group, considered changing their practice for any reduction in infection. However, the majority of surgeons [765 (80.6%)] believed that a particular irrigating solution would need to reduce the risk of infection compared to a standard by at least 25%.

**Table 7:** Need for Further Research

	**Strongly Agree**	**Agree**	**Unsure**	**Disagree**	**Strongly Disagree**
I feel there is a need for further trials to evaluate outcomes following different irrigating *solutions*	265 (28.0%)	538 (56.8%)	91 (9.6%)	47 (5.0%)	7 (0.7%)
I feel there is a need for further trials to evaluate outcomes with different irrigating *pressures*	203 (21.6%)	527 (56.0%)	144 (15.3%)	61 (6.5%)	6 (0.6%)
I feel there is a need for studies on the cost-effectiveness of different irrigating solutions	203 (21.5%)	471 (49.9%)	166 (17.6%)	93 (9.9%)	11 (1.2%)
I would change my practice if a large randomized controlled trial showed clear benefit of an irrigating solution if it was different than my current irrigating solution	504 (53.4%)	385 (40.8%)	37 (3.9%)	10 (1.1%)	8 (0.8%)

**Table 8:** Clinical Importance and Interest in Participating in Study

		**No. (%)**
Amount an alternative irrigating solution technique needs reduce infection risk before the improvement is considered "clinically important" (Relative Risk Reduction)	Any reduction at all	181 (19.3%)
	5%	100 (10.7%)
	10%	167 (17.8%)
	15%	63 (6.7%)
	20%	155 (16.5%)
	25%	99 (10.6%)
	30%	51 (5.4%)
	35%	8 (0.9%)
	40%	10 (1.1%)
	50%	57 (6.1%)
	>50%	6 (0.6%)
I would participate in a multi-centre randomized controlled study assessing different irrigating solutions and irrigating pressures	Yes	612 (62.7%)
	No	359 (36.8%)
	Maybe	5 (0.5%)
There is a need for randomized trials to evaluate irrigating solutions	Yes	803(84.7%)
I would change my practice if a trial showed a clear benefit of one irrigating solution or pressure IF it was different from my current approach	Yes	889(94.2%)

## Discussion

### Key Findings

The results of this survey demonstrated four key findings: 1) Most surgeons preferred the use of normal saline alone when irrigating open fracture wounds, 2) Surgeons felt that additives such as bacitracin and soap were either equivalent to or more effective than normal saline alone, 3) Surgeons were fairly uniform at defining low pressure but were less so when defining high pressure irrigation, and 4) most surgeons felt there was a need for further clinical trials on types of irrigating solutions as well as the pressure of irrigating solutions and would consider participating in a multi-centre randomized trial.

### Strengths and Limitations

The strengths of our study include: 1) the use of a rigorous process for the development of the questionnaire items with active surgeon participation, 2) a comprehensive sampling of surgeons of North America and Europe as well as academic and non-academic centres, and 3) an acceptable survey response rate of approximately 56% that helps to limit, but not eliminate, non-responder bias [16,17]. Our response rate of over 900 surgeons provided a robust data set for the general purposes of our study, and it exceeded the level for our anticipated study precision. Nevertheless, future studies that are aimed at more rigorously evaluating potential non-responder bias will need to institute a strategy to: 1) define non-responders by a temporal cut-off, and 2) re-administer the survey one more or more times in an attempt to achieve a sufficient level of non-responder compliance for statistical evaluation. Similar strategies have been employed in survey studies of orthopaedic surgeons' behaviors/practices on topics where responder bias would seem highly likely [18] compared to those that would seem, similar to the present study, unlikely [19].

### Irrigating Pressures

Experimental data suggests that high pressure lavage may be more effective than low pressure lavage for removing debris and bacteria from contaminated open wounds[12,13,20]. However, the efficacy in removing debris and bacteria may come at the expense of damage to bone as well as bacterial propagation into the intramedullary canal[21,22]. Cellular effects relating to the use of high pressure lavage have also been reported with an observed reduction and promotion of stem cell differentiation toward the adipocyte cell type rather than osteoblast cell type [20,23,24]. These cellular level effects have been seen to translate into a significant reduction in fracture callus strength[25]. Indeed, when undergoing mechanical testing, callus in rat femurs which had undergone high pressure irrigation of bone ends showed a significantly lower peak bending force and stiffness when compared to the control and bulb syringe (low pressure) groups (p < 0.05)[25]. The use of high pressure irrigation resulted in a 37% lower bending force compared to the low pressure irrigation and control groups[25]. However, conflicting results have been described by other investigators. While Dirschl et al., have found similar results and early detriment to fracture healing with high-pressure pulsatile lavage (HPPL), others have reported that HPPL has a similar effect on bone damage when compared with low-pressure irrigation and does not drive the bacteria further into the surrounding tissues [14,21,26].

### Irrigating Solutions

Experimental studies have evaluated several irrigation additives including antiseptics, antibiotics, and surfactants. Antiseptics have been shown to exhibit toxicity to the host cells [2,27-30]. Although antibiotics (such as bacitracin) and surfactants (such as castile soap) are routinely used for open wound irrigation, their relative effects on clinically important outcomes remain unknown. Other experimental evidence suggests that soap solution may be more effective for removing bacteria than normal saline, with a greater efficacy for removing some organisms over others [3,12,27,31,32].

In an in-vitro calvarial cell culture model, 1% soap solution has been shown to have preserved both alkaline phosphatase activity and bone nodule formation to the greatest extent when compared to other solutions (p < 0.05)[12]. Moreover, soap solution preserved osteoclast numbers to the greatest extent. In contrast, proviodine and chlorhexidine solutions resulted in the greatest decline in bone nodule formation, alkaline phosphatase activity, and osteoclast numbers (p < 0.001) [12]. We were surprised that over 40% surgeons believed proviodine was superior to normal saline given a complete lack of evidence that such antiseptics have any benefit and experimental evidence suggesting they may actually cause harm at the cellular level [12].

### Limited Clinical Evidence

There is a paucity of clinical trials evaluating open fracture wounds. Museru et al., conducted a randomized controlled trial comparing isotonic saline, distilled water and boiled water in the irrigation of open fracture wounds[33]. They reported an infection rate of 35% in the isotonic saline group, 17% in the distilled water group and 29% in the boiled water group. While statistical testing was limited, they suggested that the rates of infection were not influenced by the type of irrigating solution used[33]. Recently, Anglen conducted a large randomized trial of 400 patients with 458 open fractures has been conducted which compared castile soap and bacitracin as additives to normal saline solution[11]. The results suggested that there was no significant difference between these irrigating solutions (infection risk with soap versus bacitracin: 13% vs 18%, p = 0.2, respectively)[11]. Even though 95% confidence interval was wide (risk reduction = 28%, 95%CI: -26%–55%) the point estimate of the treatment effect displayed a trend towards the use of soap solution in reducing the risk of infection compared to antibiotic solution (28% reduction in infection risk)[11]. This lack of significance however may have been associated with the overall power of the study.

No further clinical trials have been completed comparing either normal saline alone or normal saline with soap additive in a prospective randomized fashion.

### Need for Further Clinical Trials

We have shown that there are varied opinions from surgeons on the management of open fracture wounds as regards initial irrigation and debridement. While recent clinical evidence suggests a trend towards decreased infection rates with soap solution, larger trials are needed to definitely resolve whether soap is a better alternative. Secondly, there is as yet no clinical trial on the efficacy of different irrigating pressures on outcomes such as infection rates, union rates or functional outcomes.

### Planning Another Trial

Our survey findings have aided in the planning of a pilot study. A randomized controlled trial is currently underway in pilot phase and is titled F.L.O.W (Fluid Lavage in Patients with Open Fracture Wounds). This study is a 2 × 2 factorial design with randomization into one of 4 groups: 1) Normal saline, low pressure irrigation, 2) Normal saline, high pressure irrigation, 3) normal saline/soap solution, low pressure irrigation and 4) normal saline/soap solution, high pressure irrigation. Randomization is underway in select pilot centers in both Canada, the United States and Europe, with active recruitment of centers for a large scale multicenter trial. Sample size calculations for the large trial have been based on an estimate of 20% infection in the saline control group[4]. This was calculated for a desired power of 0.80 and α = 0.05, estimating 13% and 20% rates of infection (35% Relative risk reduction) in the treatment and control groups, respectively, which represents a clinically important difference[11]. The final adjusted sample size was 536 per group, or a total of 1072 patients. Given that over 1000 patients are needed for the large trial, feasibility issues necessitate this be done in a multicenter fashion. The first 100 patients serving in the pilot study will be used to assess estimates of treatment effect and issues surrounding the sample size needed for a large trial. If infection rates are significantly lower than suggested from previous trials, the sample size will increase significantly. If we identify a gradient of effect between High and Low pressure lavage, we will also consider adding a third arm (very low pressure, bulb syringe), further increasing the study sample size to over 1500 patients. 

## Conclusion

Overall, the majority of surgeons favour both normal saline and low pressure lavage for the initial management of open fracture wounds. However, opinions varied as regards the comparative efficacy of different solutions, the use of additives and high versus low pressure. Surgeons have expressed considerable support for a trial evaluating both irrigating solutions and pressures.

## Appendix 1: FLOW investigative Team

### Steering Committee (Pilot Study)

Mohit Bhandari, MD (Hamilton, ON); Gordon Guyatt MD (Hamilton, ON); Brad Petrisor, MD (Hamilton, ON); Kyle Jeray, MD (Greenville, SC); Emil Schemitsch, MD (Toronto, ON); Stephen Walter, PhD (Hamilton, ON).

### Adjudication Committee (Pilot Study)

Mohit Bhandari MD (Hamilton, ON), Brad Petrisor MD(Hamilton, ON), Emil Schemitsch MD (Toronto, ON), and Kyle Jeray MD(Greenville, SC).

### Investigators (Pilot Study)

Dennis Beck, MD (Evansville, IN); Mohit Bhandari, MD (Hamilton, ON); Chad Coles, MD (Halifax, NS); Cory Collinge, MD (Fort Worth, TX); Joseph Conflitti, MD (Tyler, TX); Gregory Della Rocca, MD (Columbia, MO); Paul Duffy, MD (Calgary, AB); Edward Harvey, MD, (Montreal, QC); Kyle Jeray, MD (Greenville, SC); Alan Jones, MD (Dallas, TX); Sumito Kawamura, MD (Hokkaido, Japan); Kenneth Koval, MD (Lebanon, NH); Hans Kreder, MD (Toronto, ON); Yves Laflamme, MD (Montreal, QC); Theodore Miclau, MD (San Francisco, CA); Silas Motsitsi, MD (Pretoria, South Africa); Peter O'Brien, MD, (Vancouver, BC); Steve Papp, MD, (Ottawa, ON); Brad Petrisor, MD (Hamilton, ON); Laura Phieffer, MD (Columbus, OH); Robert Probe (Temple, TX); George Russell, MD (Jackson, MS); Claude Sagi, MD (Tampa, FL); Parag Sancheti, MD (Pune, India); David Sanders, MD London, ON); Emil Schemitsch, MD (Toronto, ON); Dan Schlatterer, MD (Atlanta, GA); Trevor Stone, MD (New Westminster, BC); and Bob Zura, MD (Durham, NC).

### Research Coordinators (Pilot Study)

Natalie Ansell (Hamilton, ON); Kate Babusiak (Durham, NC); Tigist Belaye (San Francisco, CA); Leah Briggs (Columbia, MO); Naomi Crnko (Toronto, ON); Carole Ehleben (Atlanta, GA); Kristy Genuario (Lebanon, NH); Chris Herrick (Temple, TX); Susie Howell (Tyler, TX); Raman Johal (Vancouver, BC); Skye Macalester (Columbus, OH); Caterina Marrazza (Montreal, QC); Tashay Mignott (Hamilton, ON); Zhen Qin (Jackson, MS); Laura Quigley (Hamilton, ON); Marie-France Poirier (Montreal, QC); Steve Rocha (Pune, India); Stephanie Tanner, MSc (Greenville, SC); Milena Santos (Toronto, ON); Christina Tieszer (London, ON); Kelly Trask (Halifax, NS); Liisa Vexler, (Ottawa, ON); and Mauri Zomar (New Westminster, BC).

### Coordinating Center (McMaster University, Hamilton, Ontario)

Natalie Ansell; Lisa Buckingham, BSc; Diane Heels-Ansdell, MSc; Christina Lacchetti, MSc; Tashay Mignott; Laura Quigley; and Sheila Sprague, MSc.

## Authors' contributions

Study Concept and Design: MB, BP. Study Coordination: SS. Data Analysis: MB, BP, SS. Drafting Manuscript: BP, MB, KJ, DS, BH, SS. Revision Manuscript: BP, MB, KJ, DS, BH, SS.

## Pre-publication history

The pre-publication history for this paper can be accessed here:


